# Supplementary material for: Focus Groups to Inform User-Centered Development of an eHealth Sleep Intervention for Adolescents: Perspectives of Youth with Insomnia Symptoms, with and without Pain
Source: Children (Basel). 2023 Oct 16;10(10):1692. doi: 10.3390/children10101692 (PMC10605192; doi:10.3390/children10101692)
Supplement: Supplementary file 1 [file children-10-01692-s001.zip › children-2587164-supplementary.pdf]

Supplementary Materials: Focus Group Discussion Guides

NOTE: The questions and probes highlighted in yellow were only asked in the pain groups. The non-highlighted questions and probes were asked at both pain and pain-free groups.

Youth Discussion Guide

NOTE: These questions are only a guide. The actual focus group discussion will be flexible and follow the participants' conversations.

Topic 1: Introductions, experience with pain (10 minutes)
Let's go through the virtual room and say our first names and briefly describe the type of pain that you experience. So for example, "My name is Michelle, and I have headaches every couple of days. Sometimes they keep me from going out and doing things with my friends."

Topic 2: Role of pain and sleep (10 minutes)
We are interested in understanding the role that sleep has in your lives.

- What problems do you have sleeping, if any?
    - *Probes: difficulty falling or staying asleep, night awakenings, early morning awakenings, short sleep duration, or poor sleep quality*
- How does pain impact your sleep?
    - *Probes: As a disruptor of sleep? A cause for sleep due to exhaustion from pain?*
- How does your sleep impact your pain?
    - *Probes: As a pain management strategy? As a risk factor for pain?*
- Do you take any pain medications that impact your sleep?
- How does pain medication impact your sleep problems?
- What strategies have you have used to address the impact that medication has had on your sleep problems?
- Are there other non-pharmacological pain-management strategies that you have impacted your sleep?
    - *Probes: Napping, resting in a dark room*
- What sleep strategies, if any, have you been recommended to try?
    - Who recommended these? *Probes: Parents, health care professionals, teachers*

Topic 3: Adapting a sleep education program for adolescents with pain (30 minutes)
Next we will discuss what youth need in terms of sleep and some strategies that are used to help youth sleep better. We want to know your ability to use these strategies.

- To have good sleep teenagers 14-17 years old need to sleep for 8-10 hours per night without any naps during the day.
    - Do you know this? Are you successful with this right now?
    - Have you tried this?
        - If you have, what got in the way, or was challenging for you?
        - If you have, and you were unsuccessful, what was the main factor that caused you to be unsuccessful with following this recommendation?

Prompts: a lack of motivation, busy schedule, worrying about school tests/assignments

- What do you do if this is a problem?
  - *Prompts: Use a daily sleep diary, get out of bed if awake for 15 minutes, address unrealistic beliefs and fears about sleep, relaxation strategies.*
- What would be motivating for you to do this?
  - *Prompts: Have a parent check in, have a friend do the same thing, be aware of long-term, or short-term consequences.*
- If you won't do it, what would be a manageable recommendation?

- How does pain interfere with your ability to sleep 8-10 hours per night without any naps, if at all?
  - How could pain be addressed to make this possible (e.g., *not taking naps*).
  - What would be motivating for you to do this?

- It is also important to have bedtimes and wake times that allow you to get enough sleep.
  - Do you know this? Do you do this now?
  - Have you tried this?
    - If you have, and you were unsuccessful, what was the main factor that caused you to be unsuccessful with following this recommendation?

      Prompts: a lack of motivation, busy schedule, worrying about school tests/assignments
  - What would be motivating for you to do this?
    - *Prompts: Have a parent check in, have a friend do the same thing, be aware of long-term, or short-term consequences.*
  - If you won't do it, what would be a manageable recommendation?

  - How does pain interfere with your ability have bedtimes and wake times that allow you to get enough sleep, if at all?
    - How could pain be addressed to make this possible (e.g., *not taking naps*).
    - What would be motivating for you to do this?

- It is also important to have consistent bedtimes and wake times for both weekdays and weekends. By this we mean no more than a 1 hour difference in bedtimes and wake times on school nights and weekend nights.
  - Do you know this? Do you do this now?
  - Have you tried this?
    - If you have, and you were unsuccessful, what was the main factor that caused you to be unsuccessful with following this recommendation?

      Prompts: a lack of motivation, busy schedule, worrying about school tests/assignments
  - What do you do if this is a problem?
    - *Prompts: Use a daily sleep diary, get out of bed if awake for 15 minutes, address unrealistic beliefs and fears about sleep, relaxation strategies.*
  - What would be motivating for you to do this?

        - *Prompts: Have a parent check in, have a friend do the same thing, be aware of long-term, or short-term consequences.*
    - If you won't do it, what would be a manageable recommendation?

    - How does pain interfere with your ability have consistent bedtimes and wake times for both weekdays and weekends, if at all?
        - How could pain be addressed to make this possible (e.g., *not taking naps*).
        - What would be motivating for you to do this?

- Establishing and following bedtime routines is also important. By this we mean doing the same things in the same order around the same times. So for example, if your bedtime is 10pm then your bedtime routine might be at 9:30 you have a small snack, 9:40 you get into your PJs and clean up (brush teeth, wash face), at 9:45 you say goodnight to your family and at 9:50 you get into bed and read for 10 min, and at 10pm you turn the lights out and try to fall asleep. Bedtime routines should be relatively short (30 mins or less), relaxing, and done consistently each night. *Note to facilitators: Tailor these time examples to bedtimes that are given by group members during earlier discussion.*
    - Do you know this? Do you do this now?
    - Have you tried this?
        - If you have, and you were unsuccessful, what was the main factor that caused you to be unsuccessful with following this recommendation?
          Prompts: a lack of motivation, busy schedule, worrying about school tests/assignments
    - What do you do if this is a problem?
        - *Prompts: Use a daily sleep diary, get out of bed if awake for 15 minutes, address unrealistic beliefs and fears about sleep, relaxation strategies.*
    - What would be motivating for you to do this?
        - *Prompts: Have a parent check in, have a friend do the same thing, be aware of long-term, or short-term consequences.*
    - If you won't do it, what would be a manageable recommendation?

    - How does pain interfere with your ability to establish and follow bedtime routines, if at all?
        - How could pain be addressed to make this possible (e.g., *not taking naps*).
        - What would be motivating for you to do this?

- There are also some things you can do to make falling asleep easier. You can change your sleep environment to be conducive with sleep. Let's explore these ideas…
    - Sleep in the same location every night
    - Bedroom should not be overly stimulating, but rather relaxing and free from distractions
    - Sleep in a dark, quiet bedroom at bedtime
    - Have no electronics (including cell phone) in your bedroom
    - Using your bed and bedroom only for sleep
        - Do you know all of these healthy sleep habits? Do you do all of these?
        - Have you tried these?

                - If you have, and you were unsuccessful, what was the main factor that caused you to be unsuccessful with following this recommendation?
                    Prompts: a lack of motivation, busy schedule, worrying about school tests/assignments
            - What do you do if this is a problem?
                - *Prompts: Use a daily sleep diary, get out of bed if awake for 15 minutes, address unrealistic beliefs and fears about sleep, relaxation strategies.*
            - Which factors seem to be most difficult for you to follow? Why?
            - What would be motivating for you to do this?
                - *Prompts: Have a parent check in, have a friend do the same thing, be aware of long-term, or short-term consequences.*
            - If you won't do it, what would be a manageable recommendation?

    - How does pain interfere with your ability to do things to make falling asleep easier, if at all?
        - How could pain be addressed to make this possible (e.g., *not taking naps*).
        - What would be motivating for you to do this?

- There are also some things you can do leading up to bedtime that make falling asleep easier. Let's explore these…
    - No electronics in the hour before bed
    - No caffeine in the few hours before bed
    - No activities that are exciting and get you energized (e.g., playing sports) in the hour before bed
    - Not going to bed hungry, or consuming a large meal right before bedtime
        - Do you know all of these healthy bedtime habits? Do you do all of these?
        - Have you tried this?
            - If you have, and you were unsuccessful, what was the main factor that caused you to be unsuccessful with following this recommendation?
                Prompts: a lack of motivation, busy schedule, worrying about school tests/assignments
        - What do you do if this is a problem?
            - *Prompts: Use a daily sleep diary, get out of bed if awake for 15 minutes, address unrealistic beliefs and fears about sleep, relaxation strategies.*
        - Which factors seem to be most difficult for you to follow? Why?
        - What would be motivating for you to do this?
            - *Prompts: Have a parent check in, have a friend do the same thing, be aware of long-term, or short-term consequences.*
        - If you won't do it, what would be a manageable recommendation?

    - How does pain interfere with your ability to do things you can do leading up to bedtime that make falling asleep easier, if at all?
        - How could pain be addressed to make this possible (e.g., *not taking naps*).

        - What would be motivating for you to do this?

- There are also some things that you can do during the day that make a good foundation for sleep
    - Eating a healthy, well balanced diet
    - Engaging in physical activity on a daily basis
        - Do you know this? Do you do this?
        - Have you tried this?
            - If you have, and you were unsuccessful, what was the main factor that caused you to be unsuccessful with following this recommendation?

              Prompts: a lack of motivation, busy schedule, worrying about school tests/assignments
        - What do you do if this is a problem?
            - *Prompts: Use a daily sleep diary, get out of bed if awake for 15 minutes, address unrealistic beliefs and fears about sleep, relaxation strategies.*
        - Which factors seem to be most difficult for you to follow? Why?
        - What would be motivating for you to do this?
            - *Prompts: Have a parent check in, have a friend do the same thing, be aware of long-term, or short-term consequences.*
        - If you won't do it, what would be a manageable recommendation?

    - How does pain interfere with your ability to do things during the day that make a good foundation for sleep, if at all?
        - How could pain be addressed to make this possible (e.g., *not taking naps*).
        - What would be motivating for you to do this?

- What other sleep strategies or considerations do you feel are important to implement for youth experiencing chronic pain?

Topic 4: Barriers to participation and strategies to overcoming barriers (10 minutes)

Next, we are interested in learning about your opinion on using a sleep intervention.

- How helpful would a sleep intervention be for you? Is this something that you would want to have available?
    - What would you hope to gain out of a sleep intervention?
- In what ways would an online intervention be more appealing than an intervention delivered in person? Less appealing?
- What do you think would stop you from using an online sleep education program?
    - What could we do to overcome those barriers?
- If the program could contain any features or content, what would be most likely to keep you using the program, and stop you from leaving?

    - What features would you recommend?
        - *Prompts: Videos, games, quizzes, tracking sleep behaviour*
    - What visuals would be most appealing?

        - *Prompts: Video style (animated vs. real-life person), colours, photos*
    - What features tend to draw you into online websites? What features keep you engaged?

- How would pain be involved in stopping you from using the program?

<u>Topic 5: Closing questions</u> (10 minutes)

- Do you have any other tips about how to make this online sleep program interesting and relevant to high school students?
- What suggestions do you have for recruiting youth with chronic pain to participate in our online intervention?
- Can you think of any other problems that might come up when providing this program?
- Is there anything we haven't touched on yet that you would like to mention? (Final question)

Stakeholder Discussion Guide

NOTE: These questions are only a guide. The actual focus group discussion will be flexible and follow the participants' conversations.

Topic 1: Introductions, child's experience with pain (10 minutes)

Let's go through the virtual room and say our first names, our roles (whether a parent or a health care professional) and briefly describe your experience with children experiencing pain. So for example, "my name is Michelle, and my daughter has headaches every couple of days. Sometimes they keep her from going out and doing things with her friends."

Topic 2: Role of pain and sleep in child's life (10 minutes)

We are interested in understanding the role that sleep has in the lives of children with pain who you have interacted with, whether as a parent, or as a health care professional.

- What problems do you find that youths with pain have for sleeping?
    - *Probes: difficulty falling or staying asleep, night awakenings, short sleep duration, early morning awakenings, or poor sleep quality*
- How does pain impact adolescents' sleep?
    - *Probes: As a disruptor of sleep? A cause for sleep due to exhaustion from pain?*
- How does sleep impact adolescents' pain?
    - *Probes: As a pain management strategy? As a risk factor for pain?*
    - Do you ever recommend sleep as a management strategy? How is this recommended?
- How does pain medication impact adolescent's sleep problems?
- What strategies do you find are helpful to address the impact that medication has had on adolescent sleep problems?
- Are there other non-pharmacological pain-management strategies that impact adolescent sleep?
    - *Probes: Napping, resting in a dark room*

Topic 3: Adapting a sleep education program for adolescents with pain (30 minutes)

Next we will discuss what youth need in terms of sleep and some strategies that are used to help youth sleep better. We want to know how you think pain impacts adolescents' sleep and their ability to use these strategies.

- To have good sleep youth need to sleep for 8-10 hours per night without any naps during the day.
    - Have you recommended this?
        - If so, what got in the way or was challenging?
    - What do you recommend if this is a problem?
        - *Prompts: Use a daily sleep diary, get out of bed if awake for 15 minutes, address unrealistic beliefs and fears about sleep, relaxation strategies*
    - Why do you think youth are having difficulty following this?
    - What do you think would be motivating for youth to do this?

        - *Prompts: Have a parent check in, have a friend do the same thing, be aware of long-term, or short-term consequences.*
    - If you don’t think this recommendation will be followed, what would be a manageable recommendation?

    - How does pain interfere with youth’s ability to sleep for 8-10 hours per night without any naps during the day, if at all?
        - How could pain be addressed to make this possible (e.g., *not taking naps*).
        - What would be motivating for youth to do this?

- It is also important to have bedtimes and wake times that allow youth to get enough sleep.
    - Have you recommended this?
        - If so, what got in the way or was challenging?
    - What do you recommend if this is a problem?
        - *Prompts: Use a daily sleep diary, get out of bed if awake for 15 minutes, address unrealistic beliefs and fears about sleep, relaxation strategies*
    - Why do you think youth are having difficulty following this?
    - What do you think would be motivating for youth to do this?
        - *Prompts: Have a parent check in, have a friend do the same thing, be aware of long-term, or short-term consequences.*
    - If you don’t think this recommendation will be followed, what would be a manageable recommendation?

    - How does pain interfere with youth’s ability to have bedtimes and wake times that allow youth to get enough sleep, if at all?
        - How could pain be addressed to make this possible?
        - What would be motivating for youth to do this?

- It is also important to have consistent bedtimes and wake times for both weekdays and weekends. By this we mean no more than a 1 hour difference in bedtimes and wake times on school nights and weekend nights.
    - Have you recommended this?
        - If so, what got in the way or was challenging?
    - What do you recommend if this is a problem?
        - *Prompts: Use a daily sleep diary, get out of bed if awake for 15 minutes, address unrealistic beliefs and fears about sleep, relaxation strategies*
    - Why do you think youth are having difficulty following this?
    - What do you think would be motivating for youth to do this?
        - *Prompts: Have a parent check in, have a friend do the same thing, be aware of long-term, or short-term consequences.*
    - If you don’t think this recommendation will be followed, what would be a manageable recommendation?

    - How does pain interfere with youth’s ability to have consistent bedtimes and wake times for both weekdays and weekends, if at all?

        - How could pain be addressed to make this possible?
        - What would be motivating for youth to do this?

- Establishing and following bedtime routines is also important. By this we mean doing the same things in the same order around the same times. So for example, if a youth's bedtime is 10pm then the youth's bedtime routine might be at 9:30 they have a small snack, 9:40 they get into their PJs and clean up (brush teeth, wash face), at 9:45 they say goodnight to their family and at 9:50 they get into bed and read for 10 min, and at 10pm they turn the lights out and try to fall asleep. Bedtime routines should be relatively short (30 min or less), relaxing, and done consistently each night.
    - Have you recommended this?
        - If so, what got in the way or was challenging?
    - What do you recommend if this is a problem?
        - *Prompts: Use a daily sleep diary, get out of bed if awake for 15 minutes, address unrealistic beliefs and fears about sleep, relaxation strategies*
    - Why do you think youth are having difficulty following this?
    - What do you think would be motivating for youth to do this?
        - *Prompts: Have a parent check in, have a friend do the same thing, be aware of long-term, or short-term consequences.*
    - If you don't think this recommendation will be followed, what would be a manageable recommendation?

    - How does pain interfere with youth's ability to establish and follow bedtime routines, if at all?
        - How could pain be addressed to make this possible?
        - What would be motivating for youth to do this?

- There are also some things that youth can do to make falling asleep easier. Youth can change their sleep environment to be conducive with sleep. Let's explore these ideas…
    - Sleep in the same location every night
    - Bedroom should not be overly stimulating but rather relaxing and free from distractions
    - Sleep in a dark, quiet bedroom at bedtime
    - Have no electronics (including cell phone) in their bedroom
    - Using their bed and bedroom only for sleep
        - Have you recommended this?
            - If so, what got in the way or was challenging?
        - What do you recommend if this is a problem?
            - *Prompts: Use a daily sleep diary, get out of bed if awake for 15 minutes, address unrealistic beliefs and fears about sleep, relaxation strategies*
        - Why do you think youth are having difficulty following this?
        - What do you think would be motivating for youth to do this?
            - *Prompts: Have a parent check in, have a friend do the same thing, be aware of long-term, or short-term consequences.*

        - If you don't think this recommendation will be followed, what would be a manageable recommendation?

    - How does pain interfere with youth's ability to do things that can make falling asleep easier, if at all?
        - How could pain be addressed to make this possible?
        - What would be motivating for youth to do this?

- There are also some things youth can do leading up to bedtime that make falling asleep easier, let's explore these…
    - No electronics in the hour before bed
    - No caffeine in the few hours before bed
    - No activities that are exciting and get youth energized (e.g., playing sports) in the hour before bed
    - Not going to bed hungry, or consuming a large meal right before bedtime
    - Being relaxed and calm before bed
        - Have you recommended this?
            - If so, what got in the way or was challenging?
        - What do you recommend if this is a problem?
            - *Prompts: Use a daily sleep diary, get out of bed if awake for 15 minutes, address unrealistic beliefs and fears about sleep, relaxation strategies*
        - Why do you think youth are having difficulty following this?
        - What do you think would be motivating for youth to do this?
            - *Prompts: Have a parent check in, have a friend do the same thing, be aware of long-term, or short-term consequences*.
        - If you don't think this recommendation will be followed, what would be a manageable recommendation?

    - How does pain interfere with youth's ability to do things leading up to bedtime that make falling asleep easier, if at all?
        - How could pain be addressed to make this possible?
        - What would be motivating for youth to do this?

- There are also some things that youth can do during the day that make a good foundation for sleep
    - Eating a healthy, well balanced diet
    - Engaging in physical activity on a daily basis
        - Have you recommended this?
            - If so, what got in the way or was challenging?
        - What do you recommend if this is a problem?
            - *Prompts: Use a daily sleep diary, get out of bed if awake for 15 minutes, address unrealistic beliefs and fears about sleep, relaxation strategies*
        - Why do you think youth are having difficulty following this?
        - What do you think would be motivating for youth to do this?

            - *Prompts: Have a parent check in, have a friend do the same thing, be aware of long-term, or short-term consequences.*
        - If you don't think this recommendation will be followed, what would be a manageable recommendation?

    - How does pain interfere with youth's ability to do things during the day that make a good foundation for sleep, if at all?
        - How could pain be addressed to make this possible?
        - What would be motivating for youth to do this?

- What other strategies or considerations do you feel are important to implement in consideration that the population will be experiencing chronic pain?

<u>Topic 4: Barriers to participation and strategies to overcoming barriers</u> (10 minutes)
Next, we are interested in learning about your opinion on using a sleep intervention for youth with chronic pain.

- How helpful would a sleep intervention be for youth with pain? Is this something that you would want to have available? Is this something that you would recommend?

    - What would you hope for the youth to gain out of a sleep intervention?
    - What would prevent you from recommending a sleep intervention?
- In what ways would an online intervention be more appealing than an intervention delivered in person? Less appealing?
- What do you think would stop youth from using an online sleep education program?
    - How would pain be involved in stopping youth from using the program?
    - What could we do to overcome those barriers?
- If the program could contain any features or content, what would be most likely to keep youth using the program, and stop youth from leaving?
    - What features would you recommend?
        - *Prompts: Videos, games, quizzes, tracking sleep behaviour*
    - What visuals would be most appealing?
        - *Prompts: Video style (animated vs. real-life person), colours, photos*
    - What features tend to draw youth into online websites? What features keep youth engaged?

<u>Topic 5: Closing questions</u> (10 minutes)
- Do you have any other tips about how to make this online sleep program interesting and relevant to high school students?
- What suggestions do you have for recruiting youth with chronic pain to participate in our online intervention?
- Can you think of any other problems that might come up when providing this program?
- Is there anything we haven't touched on yet that you would like to mention? (Final question
